# Intellectual and Developmental Disability: Healthcare Financing

**DOI:** 10.3389/fpubh.2014.00160

**Published:** 2014-09-24

**Authors:** David A. Ervin, Joav Merrick

**Affiliations:** ^1^The Resource Exchange, Colorado Springs, CO, USA; ^2^National Institute of Child Health and Human Development, Jerusalem, Israel; ^3^Health Services, Division for Intellectual and Developmental Disabilities, Ministry of Social Affairs and Social Services, Jerusalem, Israel; ^4^Division of Pediatrics, Hadassah Hebrew University Medical Center, Jerusalem, Israel; ^5^Kentucky Children’s Hospital, University of Kentucky College of Medicine, Lexington, KY, USA; ^6^Center for Healthy Development, School of Public Health, Georgia State University, Atlanta, GA, USA

**Keywords:** intellectual and developmental disability, healthcare Financing, World Health Organization, health care, neurodevelopmental and related disabilities

## Introduction

The World Health Organization (WHO) estimates global spending on healthcare at $6.5 trillion, approximately 10.5% of the world’s gross domestic product. The United States’ (US) share of that spending is $2.6 trillion, essentially quadrupling since 1980. The 2010 United States Patient Protection and Affordable Care Act (PPACA), also known as Obamacare, has stimulated extensive debate over the way in which healthcare is financed, and whether or not the costs of healthcare are sustainable. Among publicly funded healthcare in the US, Medicaid and Medicare are primary sources of funding. In federal fiscal year 2012, Medicaid spending on acute health exceeded $275.4 billion, while a further $122.7 billion expended in Medicaid long term services and supports. The impact of an aging population worldwide (the so-called “wave of wisdom”), as the Baby Boomer generation reaches senior status, and attendant increases in chronic conditions, will be a substantial driver of healthcare costs in the future.

Among people with intellectual and developmental disabilities (IDD), cost estimates vary depending on a range of factors. Some children with IDD, for example, are covered for at least some healthcare needs by private insurance policies held by their parents, while other children and most adults with IDD rely heavily on Medicaid and, to a lesser extent, Medicare and other publicly financed healthcare options. In many US states, certain nursing and home health services are presumed to be part of funding of home and community-based service (HCBS) medicaid waiver services (typically considered part of residential service reimbursements under these waivers), and rely on medicaid state plans for other acute health services. There are also wide ranges of estimates of uncompensated care that, when combined with other variables that are difficult to control, make accurate aggregate cost estimates difficult. Birenbaum and Cohen (1) offer a review of healthcare utilization and costs in general for people with IDD.

## Financing Healthcare for this Population

There are a number of factors that are likely to contribute to the costs of healthcare for people with IDD, which will require thoughtful analyses as financing systems continue to evolve. People with IDD are living longer and experiencing declines in health that ordinarily accompany the aging process. The life expectancy for people with IDD is similar to that of the general population, with the mean age at death ranging from the mid-50s (for those with more severe disabilities or Down syndrome) to the early 70s for adults with mild to moderate IDD (2, 3). The number of adults with IDD aged 60 years and older is expected to reach 1.2 million by 2030. This increase in the lifespan of individuals with developmental disabilities will inevitably drive healthcare costs for the foreseeable future.

There are also certain chronic conditions that frequently are found among people with IDD that are known drivers of healthcare costs. In a 2012 study of nearly 1,000 adults with IDD in the US, two thirds had two or more co-morbidities, including obesity and chronic mental health needs. More than 40% of these adults were diagnosed with four or more chronic conditions in addition to IDD (4).

While we recognize these challenges to delivering high-quality and affordable, sustainable healthcare to people with IDD, we must also acknowledge that obstacles to accessing the level of care required of these particular circumstances persist and contribute to a higher cost of healthcare. To the extent that barriers prevent people with IDD accessing health promotion resources and preventative healthcare through primary care, conditions are left unaddressed until they require far more extensive and expensive interventions.

## New and Emerging Models

Models that address the costs of healthcare for people with IDD, while achieving high-quality care and outcomes are emerging. States and practitioners alike are recognizing the need to change systems of care – and, importantly, people with IDD and their advocates are pushing for greater access with better outcomes. A Center for Health Care Strategies survey (5) sent to state IDD agency directors in 2011 yielded interesting results. Among responding states, 62% indicated their intention to integrate medical and behavioral health services with their long term services and supports systems. In addition, Michigan announced plans to develop a Medicaid Chronic Care Health Home Project, a demonstration project that is intended to improve efficiency and effectiveness of healthcare and reduce health care costs. Patient-centered medical homes (or health homes) are primary healthcare practices that provide comprehensive whole-person care to those they serve: children, adults, seniors, and families, including recommended preventative care and chronic disease management. Medical homes offer coordinated care across the healthcare system, including specialists, hospitals, long term service systems, and patients’ natural support communities. These important initiatives suggest states’ recognition of the need to innovate and make more efficient their healthcare delivery and financing systems. Bachman et al. (6) provided a good overview of US state-based publicly financed healthcare options.

There are also important opportunities for healthcare delivery systems to be built on a federally qualified health center (FQHC) platform, where reimbursements are cost-based (versus fee-for-service). FQHCs qualify for enhanced reimbursement from Medicare and Medicaid, as well as other benefits. FQHCs must serve an underserved area or population, offer a sliding fee scale, provide comprehensive services, have an ongoing quality assurance program, and have a governing board of directors. Most metropolitan areas of the US are supported by FQHCs, thus representing a reasonably accessible means of healthcare for people with IDD. This does not guarantee that an FQHC will employ providers who are expert in the treatment of people with IDD; however, to the extent such expertise can be developed within an FQHC, it is a promising opportunity to overcome some of the financially driven access barriers for people with IDD.

Leadership education in neurodevelopmental and related disabilities (LEND) programs offer another highly regarded model that provides, among other things, interdisciplinary care intended to improve the health of people with IDD. These programs, which are typically a component of a university center for excellence in developmental disabilities education, research and service (UCEDD), work in collaboration with local university hospitals or health care centers. While each LEND program is unique, they share the following objectives:

- advancing the knowledge and skills of all child health professionals to improve healthcare delivery systems for children with IDD
- providing high-quality interdisciplinary education that emphasizes the integration of services from state and local agencies and organizations, private providers, and communities
- providing health professionals with skills that foster community-based partnerships and
- promoting innovative practices to enhance cultural competency, family-centered care, and interdisciplinary partnerships.

The LENDs are federally funded under the 2006 Combating Autism Act and are administered by the Health Resources and Service’s Administration’s Maternal and Child Health Bureau of the US government (7). There are fewer than 50 LEND programs in the United States.

Managed care also holds promise as a vehicle for healthcare delivery alternatives. In particular, Medicaid managed care, reviewed as long ago as 15 years ago in the context of healthcare and long term services and support for people with IDD (8), is finding increasing popularity as a way to manage healthcare financing. In the last decade, as budget pressures have mounted, US healthcare financing policy has shifted toward enrolling Medicaid beneficiaries with disabilities into managed care. Medicaid managed care, in addition to promising more efficient use of finite healthcare financing, also offers the potential for improvements in coordination of care for Medicaid enrollees, particularly those with complex needs (9). Medicaid managed care does not come without some controversy. Concerns from people with IDD, their families, and advocates focus on quality of care and who is making decisions on what healthcare can be accessed. Such concerns are consistent with broad historical perceptions that managed care systems deny necessary care and provide lower quality care (10).

Beyond these models, there are additional considerations. With PPACA in the United States, it is required that individual and small private insurance group plans cover certain benefits that have previously not been widely available, “rehabilitative and habilitative services and devices.” While private health plans have traditionally covered rehabilitative services that are designed around recovery from a traumatic health event, the health law now requires coverage of similar services to help people learn or maintain functional skills. This important advancement in coverage standards holds substantial promise for people with IDD. In individual states over the past ten years, there have been concerted efforts in state legislatures to carve out and mandate certain private insurance coverage – particularly for therapies such as applied behavior analysis for children with Autism – to support people with IDD and their families in accessing the healthcare they need to thrive.

## Conclusion

The high cost of healthcare for people with IDD is not new. People with IDD are far more likely than their typically developing peers to experience health disparities, disproportionally higher rates of chronic disease, and less access to preventative primary care and health promotion resources. The result is that people with IDD tend to access the healthcare system at a point when care and treatment will be more extensive and therefore more costly. This need not be the case. As healthcare financing systems continue to evolve, more should be done to link them to both preventative healthcare and health promotion needs and identified priority outcomes of people with IDD:

- Care coordination that considers acute health needs and service, long term service and supports, and a person’s array of natural and community supports remains a needed service for people with IDD to fully address complex healthcare conditions that require services from multiple providers and specialty types. A feature of Medicaid managed care systems, care coordination supports people with IDD and their families in accessing and receiving needed healthcare from a potentially expansive range of healthcare outlets, while promoting a more cost-effective and efficient use of healthcare resources. Consistent with this needed focus on care coordination, expansion of the health home model (11) for people with IDD should occur.
- As envisioned in the PPACA, healthcare reimbursements (both public and private) should be linked with patient outcomes. Fee-for-service systems have created incentives for healthcare providers to perform treatments and procedures, versus for health outcomes experienced by people. In addition, capitation or universal payments for healthcare that are directed, to the fullest extent possible by people with IDD and their families, should characterize healthcare financing systems of the future that allow them to actively participate in deciding how and from where they will receive their healthcare.
- Existing systems of healthcare delivery, including FQHCs and LEND programs, should be leveraged more extensively. With FQHCs, cost-based reimbursements are superior to traditional fee-for-service funding; and, it is well within the US federal guidelines for FQHCs to serve people with IDD to the extent they are “an underserved population.”
- As in 26 states in the US, work to create statutorily mandated benefits that assure children and adults covered by private insurance have access to essential services should continue. While the recent trend has been toward mandated benefits or insurance reform targeting autism (12, 13), a more expansive view of benefits that are linked to desired health outcomes for people with all forms of IDD should be taken. Moreover, these efforts, which are best led by people with IDD and their families and advocates, should be sustained over time to assure that mandated benefits packages are informed by contemporary research and best practice.

## Conflict of Interest Statement

The authors declare that the research was conducted in the absence of any commercial or financial relationships that could be construed as a potential conflict of interest.
